# Supplementary material for: Nonthermal acceleration of protein hydration by sub-terahertz irradiation
Source: Nat Commun. 2023 May 22;14:2825. doi: 10.1038/s41467-023-38462-0 (PMC10203368; doi:10.1038/s41467-023-38462-0)
Supplement: Supplementary file 1 — Supplementary Information [file 41467_2023_38462_MOESM1_ESM.pdf]

Supplementary Information

Nonthermal acceleration of protein hydration by sub-terahertz irradiation

Jun-ichi Sugiyama[1#], Yuji Tokunaga[2#], Mafumi Hishida[3,6#], Masahito Tanaka[4], Koh Takeuchi[2], Daisuke Satoh[4] and Masahiko Imashimizu[5]*

*Correspondence: m.imashimizu@aist.go.jp

[#]Contributed equally

This PDF file includes:

Supplementary Notes

Supplementary Figures 1 to 10

Supplementary Tables 1 to 2

Supplementary Notes

Sensitive detection of the changes in dielectric properties by modulating a standing wave

As described in the main text, we used Δr as an index of the deviation of the measured complex permittivity from the value calculated from the Debye relaxation model. Evaluating $P_{\Delta r}$ allowed us to sensitively capture the change in dielectric permittivity of the liquid sample due to 0.1 THz irradiation (Supplementary Fig. 1a), based on the measurement within the frequency range of 100 MHz–14 GHz. A hypothetical mechanism, through which $P_{\Delta r}$ is connected with the decrease in the dielectric permittivity of the sample, is illustrated in Supplementary Fig. 1b. In the normal reflection method, a sample path length of $l > 5$ mm satisfies the requirement that the path of an electric field is filled with the sample to infinity, wherein the applied electric field strength is negligible at the interface of the container. Therefore, the reflection occurs within the sample but not from the interface or interior of the container during measurement (Supplementary Fig. 1b). By contrast, because of the short path length of l =1.0 mm used in our method, unattenuated electromagnetic signals should be reflected at the interface of the container, which is further reflected at another interface of the open-ended coaxial probe, thereby undergoing multiple reflections (Supplementary Fig. 1b). Polydimethylsiloxane (PDMS) has a significantly lower permittivity than water, causing a strong reflection of the applied field at the interface. An l of 1.0 mm is approximately equal to λ/4 for 7–8 GHz electromagnetic waves, where λ is the electrical length through liquid water (closely approximated by the lysozyme solution) at room temperature. Therefore, we inferred that a standing wave (i.e., a λ/4 resonance of the multiply reflected electromagnetic waves) was the electrical origin of $P_{\Delta r}$, where l = 1.0 mm was not closely finetuned and slightly longer than λ/4, and consequently needed to be shortened to generate a sharp standing wave signal (Supplementary Fig. 1c). Because the standing wave is determined by l and λ, its finetuning can be achieved not only by decreasing l but also by increasing λ from the original condition (Supplementary Fig. 1c). The latter can be achieved by decreasing the dielectric permittivity of the liquid sample according to $\lambda \propto 1/\sqrt{\varepsilon}$.

Note that the actual λ /4 of 7–8 GHz electromagnetic waves through liquid water at room temperature is slightly longer than the l of 1.0 mm (λ ≈ 1.2 and 1.3 mm at 7 and 8 GHz, respectively), which appears to contradict the above explanation. This may be because either or both interfaces at which the reflection of the applied electric field actually occurred were located slightly further inside from the interfaces measured by physical contact.

To verify the validity of the aforementioned explanation, we performed similar dielectric measurements by varying l by ± 0.1 mm from the initial length of 1.0 mm, and $P_{\Delta r}$ was evaluated as a function of frequency (Supplementary Fig. 2a). Roughly, values of l longer than 1.0 mm elevated the Δr signal at ~6 GHz, whereas values shorter than 1.0 mm elevated the signal at ~8–9 GHz; the relationship between the two measurements with longer and shorter l was nearly line-symmetric at the ~7 GHz frequency position (Supplementary Fig. 2a). The Δr profiles obtained for shortening and lengthening l by 0.2 mm from 1.0 mm are positively and negatively correlated with the profile after 0.1 THz irradiation (l = 1.0 mm), respectively (Supplementary Figs. 2b and c). This result is consistent with the aforementioned explanation, i.e., identical values of $P_{\Delta r}$ can be obtained by finetuning the signal by decreasing l or increasing λ (decreasing ε).

Next, we performed a dielectric measurement of a wider frequency region (1 MHz–40 GHz) to more precisely determine the relationship between l and the frequency of $P_{\Delta r}$ (Supplementary Fig. 3). In this measurement, we changed l from 0.5 to 1.9 mm for the Unknown measurement and fixed $l > 5.0$ mm for the Standard measurement to avoid any reflection from the PDMS interface during calibration. The findings showed that the frequency of $P_{\Delta r}$, which varies with l, closely resembles the frequency dependence of $\lambda/4$ (Supplementary Fig. 3), which supports the hypothesis that $l = \lambda/4$ is the optimal condition for generating $P_{\Delta r}$. However, while not affecting the essential part of the interpretation, we could not determine the partial incompatibility of the aforementioned relationship, particularly at ~7–10 GHz, and why it depended on the coaxial probe used (Supplementary Fig. 3). The result is possibly incompletely expressed using solely the single-mode standing wave, and $l = \lambda/4$ is the dominant but not exclusive condition for the creation of the resonant signal in the measurement system.

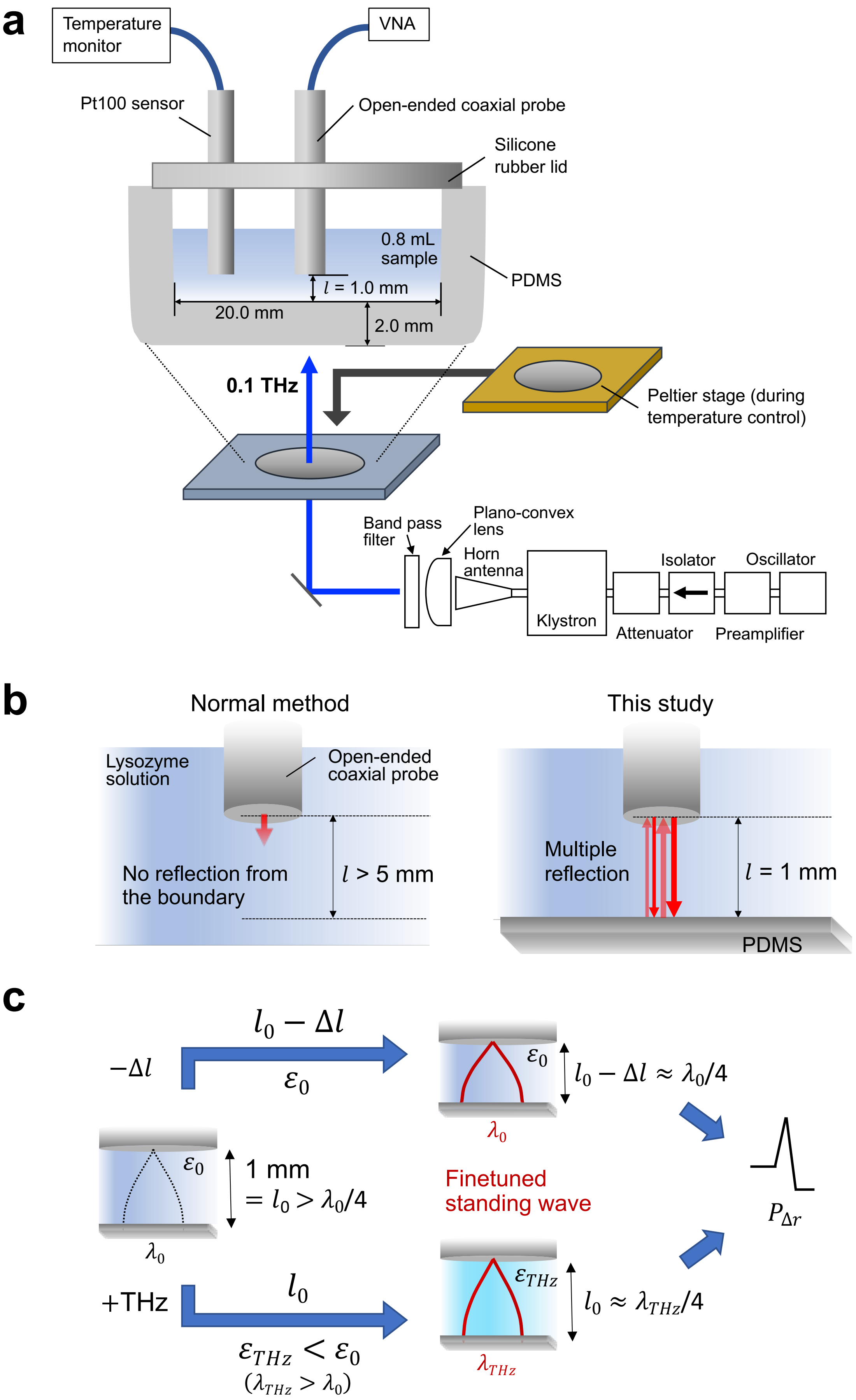
a
Temperature monitor
VNA
Pt100 sensor
Open-ended coaxial probe
Silicone rubber lid
0.8 mL sample
l = 1.0 mm
PDMS
20.0 mm
2.0 mm
0.1 THz
Peltier stage (during temperature control)
Band pass filter
Plano-convex lens
Horn antenna
Klystron
Isolator
Oscillator
Attenuator
Preamplifier
b
Normal method
This study
Lysozyme solution
Open-ended coaxial probe
No reflection from the boundary
l > 5 mm
Multiple reflection
l = 1 mm
PDMS
c
$l_0 - \Delta l$
$-\Delta l$
ε_0
$l_0 - \Delta l \approx \lambda_0/4$
λ_0
1 mm
$= l_0 > \lambda_0/4$
Finetuned standing wave
$P_{\Delta r}$
l_0
+THz
$\varepsilon_{THz} < \varepsilon_0$
$(\lambda_{THz} > \lambda_0)$
ε_{THz}
$l_0 \approx \lambda_{THz}/4$
λ_{THz}

Supplementary Fig. 1. Experimental setup and physical origin of $P_{\Delta r}$. (**a**) In situ dielectric relaxation (DR) measurement. The 0.1 THz wave was directed toward the bottom of a thin aqueous sample. Dielectric and temperature probes were connected to a vector network analyzer (VNA) and Peltier controller, respectively. A PDMS container was used as the sample cell to exploit the high adiabaticity and sub-terahertz transmittance. (**b**) Difference in dielectric measurement between the normal reflection method and this study. In the normal method assuming a path length of $l > 5.0$ mm, the applied 7-GHz electric field through water is attenuated to $< 1/100$ of the incident intensity at the bottom boundary. In this study, $l =$ 1.0 mm; consequently, the same field is attenuated to $\sim 1/e$ of its incident intensity at the boundary. (**c**) Increase in electrical length λ due to the decrease in dielectric permittivity ε can finetune a standing wave (λ /4 resonance) similarly to shortening l, both of which lead to the generation of peak of the Δr signal $P_{\Delta r}$.

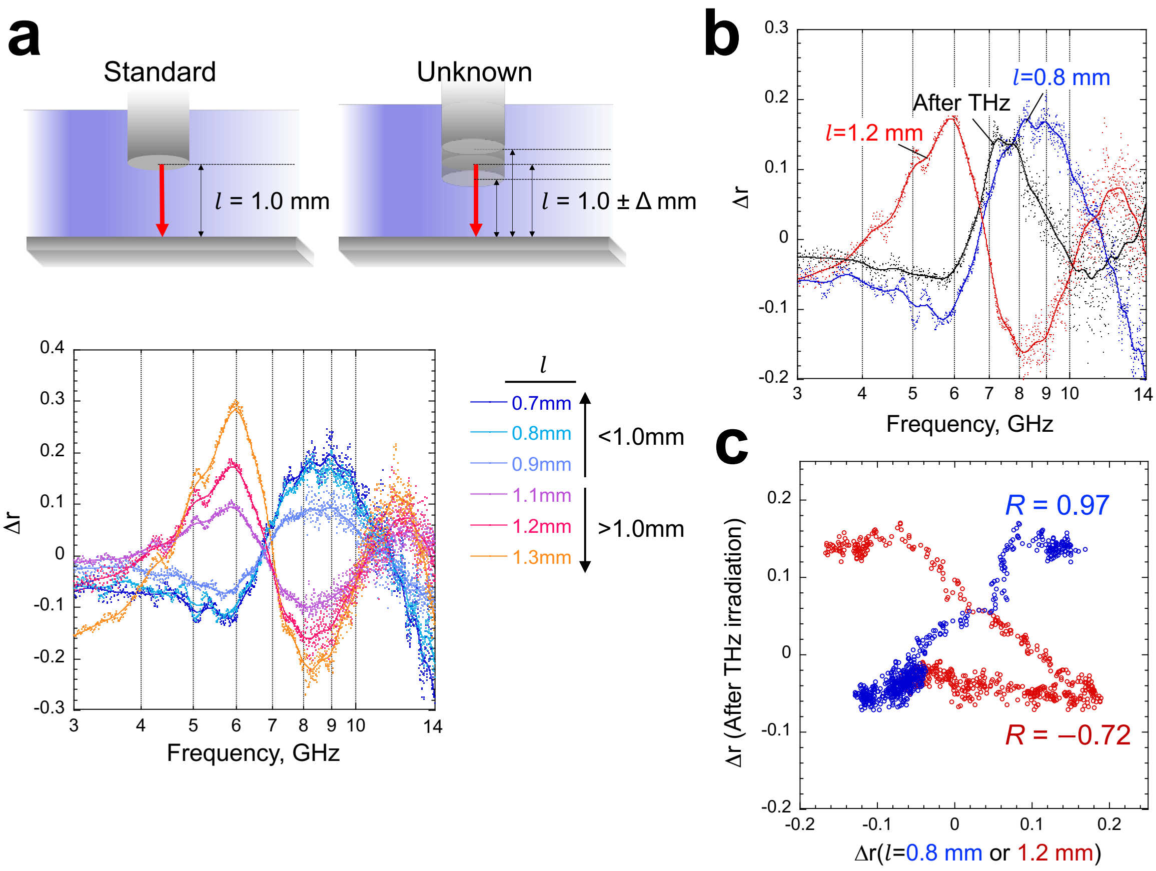

Supplementary Fig. 2. Experimental results verifying the hypothetical model illustrated in Supplementary Fig. 1c. (**a**) Mismatch ($\pm\Delta$) of the sample path length (l) between the Standard and Unknown samples in ± 0.1 mm increments (top) results in sharp changes in Δr

(deviation of measured values from the Debye model) in 3-14 GHz (bottom). (**b** and **c**) Δ*r* profile after 0.1 THz irradiation (Supplementary Fig. 5b, *t* = 60 min) correlates positively and negatively with decreasing (*l* = 0.8 mm) and increasing (*l* = 1.2 mm) sample path length, respectively. Data are shown with a moving average curve. (**c**) Δ*r* correlations for the *l*-dependent (*l* = 0.8 mm or 1.2 mm) and irradiation-dependent effects. Correlation coefficients (*R*) between the two variables are shown. In both cases, *l* = 0.8 mm and *l* =1.2 mm, the *p*-values are lower than 1×10^{-10}. Δ*r* data in the frequency range of 3–8 GHz were used for the correlation analysis because of the enhanced peak broadening observed in the > 8 GHz region for the Δ*r* profile upon varying *l* (values plotted on *x*-axis).

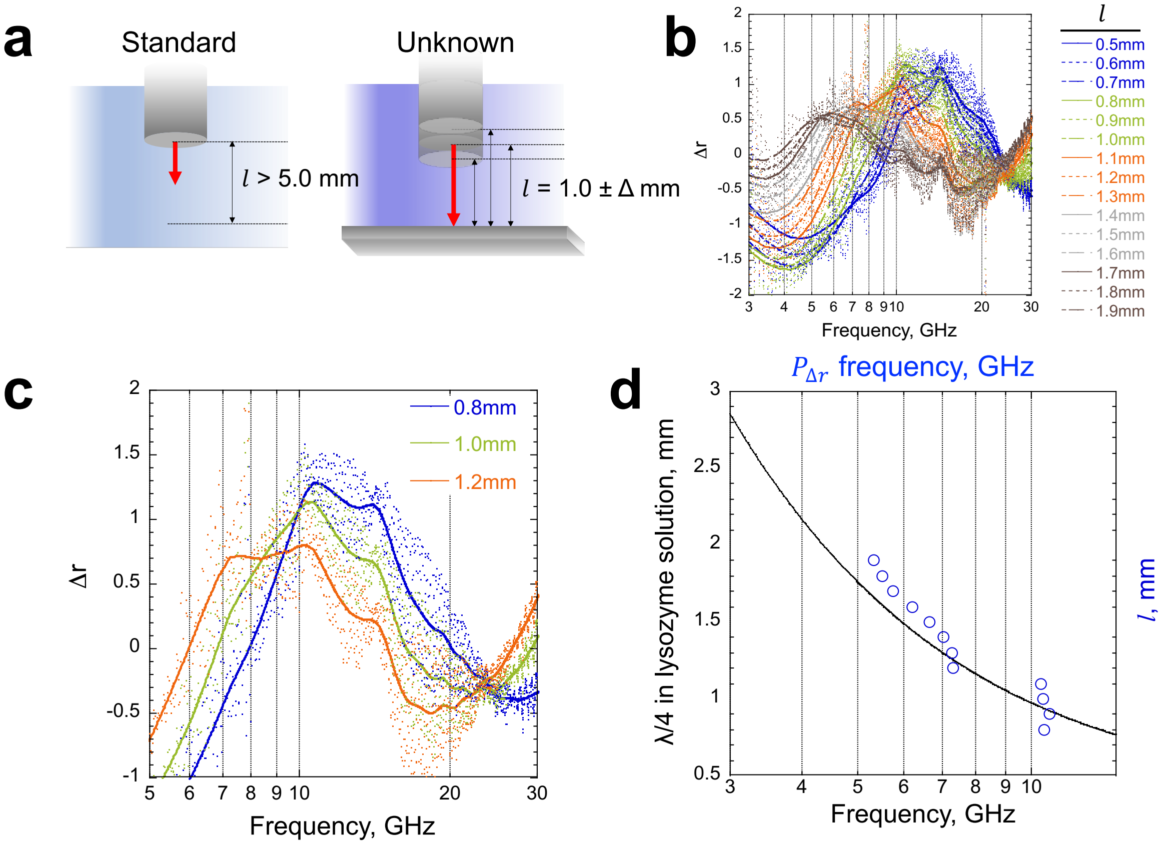

Supplementary Fig. 3. Experimental results verifying the predicted standing wave patterns. (a and **b)** DR of the Unknown sample (i.e., lysozyme solution, sample path length *l* = 0.5–1.9 mm) was compared to that of the Standard sample (pure water, *l* > 5.0 mm) in the 1 MHz–40 GHz frequency region using the VNA (MS46131A, Anritsu) and coaxial probe (N1501A Dielectric Probe Kit, Keysight). Data are shown with a moving average curve. (**c**)

Samples between l = 0.8 mm and l = 1.2 mm extracted from Panel b. (**d**) l dependence of $P_{\Delta r}$ (peak of the Δr signal) frequency agrees closely with the frequency dependence of $\lambda/4$.

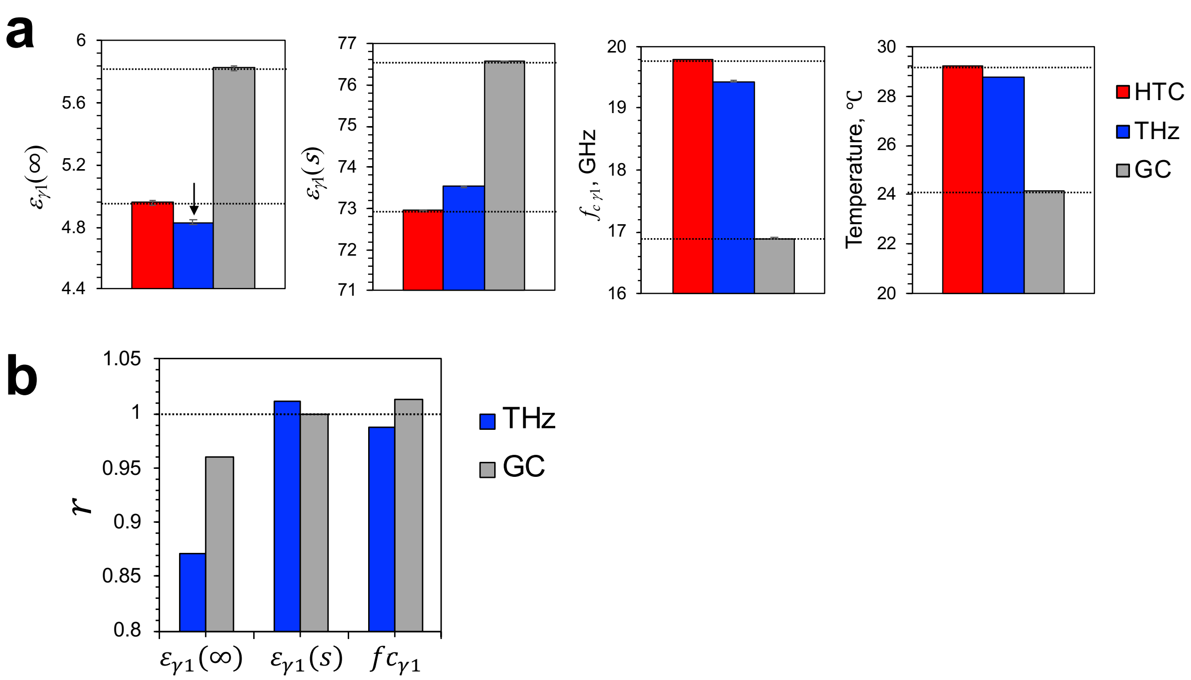

Supplementary Fig. 4. Changes in dielectric parameters of the 2.9 wt% lysozyme solution. (**a**) DR measurements at frequencies ranging from 100 MHz to 14 GHz or (**b**) 40 GHz during 0.1 THz irradiation. (**a**) Means of five measurements $\pm$ standard deviations are shown. HTC and GC values are indicated by dashed lines. The 0.1-THz-induced decrease in the high-frequency limit of dielectric permittivity of slow water $\varepsilon_{\gamma1}(\infty)$ is indicated by an arrow. (**b)** Each parameter calculated by the measurement up to 40 GHz was normalized by that calculated by the measurement up to 14 GHz, which is shown as r. When $r = 1$, the two measurements are identical (dashed line). By expanding the measurement frequency from 14 GHz to 40 GHz (i.e., to the higher frequency side of the relaxation frequency of slow water $fc_{\gamma1}$), the reduction effect of $\varepsilon_{\gamma1}(\infty)$ by 0.1 THz irradiation became larger. Because $\varepsilon_{\gamma1}(\infty)$ as an extrapolated value should become more accurate if the measurement range is expanded to the higher frequency, this result supports that the decrease in $\varepsilon_{\gamma1}(\infty)$ by irradiation is not an artifact due to the narrow measurement range.

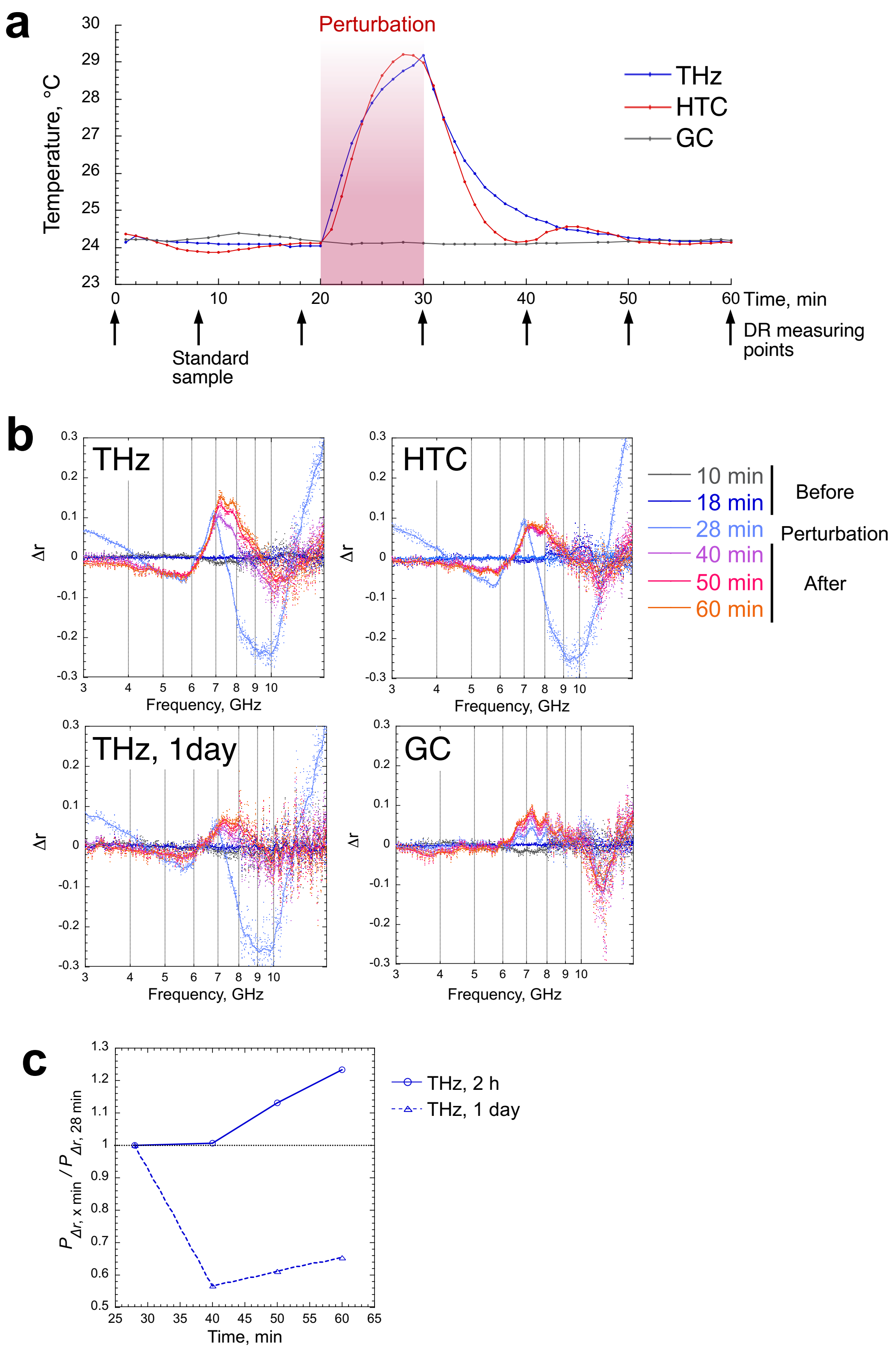

Supplementary Fig. 5. Effect of 0.1 THz irradiation on the 2.9 wt% lysozyme solution is qualitatively identical to that on the 9.1 wt% solution. (**a**) Time course of the DR measurements of 2.9 wt% lysozyme solution with different perturbations. Temperatures measured in real time in the corresponding experiments are represented on the *y*-axis. (**b**) Changes over time in the Δr (deviation of measured values from the Debye model) profiles of

2.9 wt% lysozyme solution for 0.1 THz irradiation (THz), high-temperature control (HTC), and general control with a constant temperature (GC). The means of five measurements and moving average curves are shown. For THz, the results 2 h (top) and 1 day (bottom) after water dissolution of the samples are shown. (**c**) Increase in $\boldsymbol{P}_{\Delta r}$ (peak of the Δr signal) due to 0.1 THz irradiation is dependent on the time from water dissolution of the sample. $\boldsymbol{P}_{\Delta r}$ is given by subtracting the baseline (mean of Δr at each 1 GHz region on both sides of the peak).

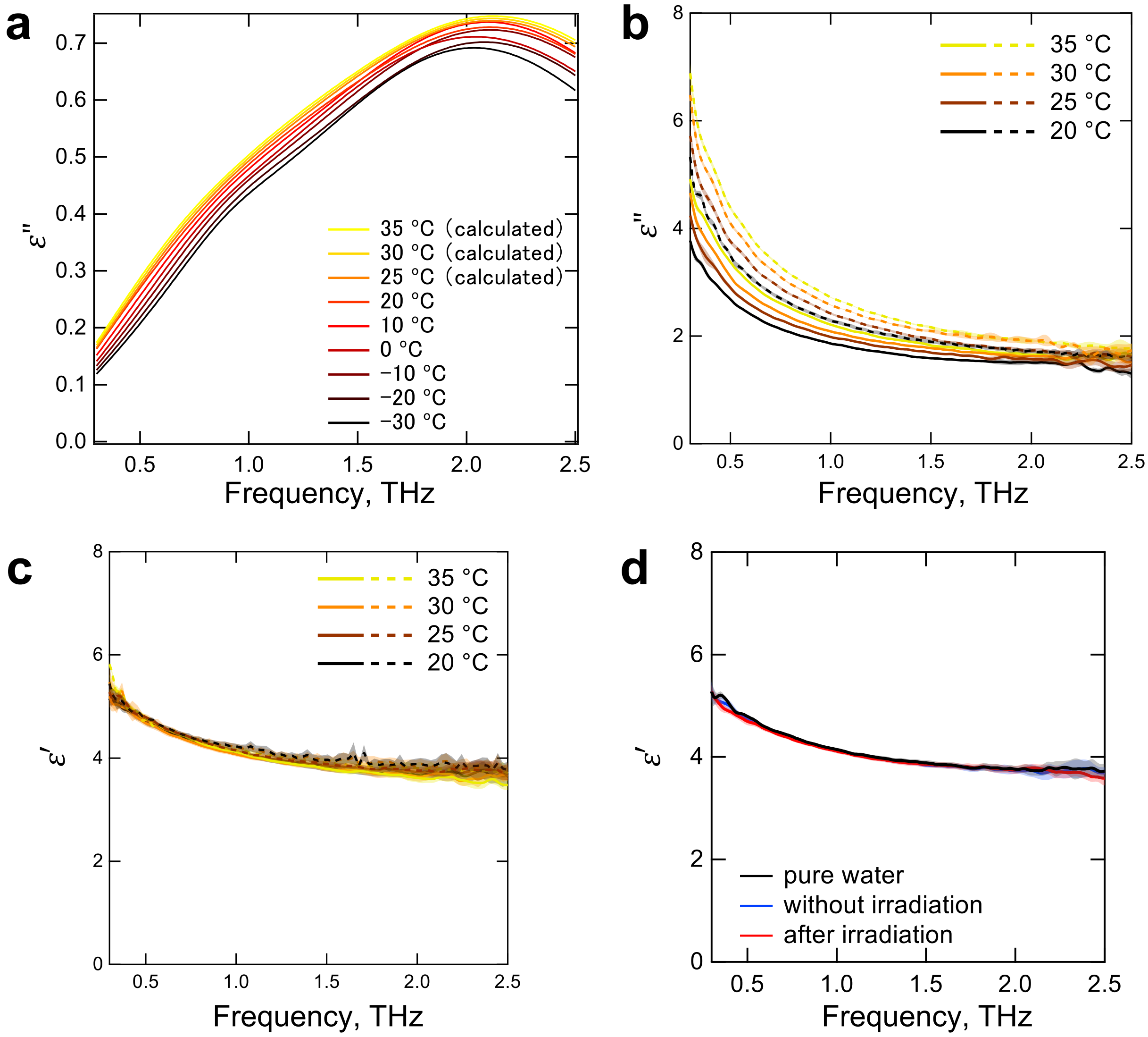

Supplementary Fig. 6. Analysis for THz-TDS measurements. (a) Spectra for dehydrated lysozyme [4]. Spectra for 25–35 °C were calculated by linear extrapolation of those of −30–20 °C. (**b**) Spectra of 28.6 wt% lysozyme solution (solid lines) and those of pure water

(dashed lines) at different temperatures. (**c**) Real part of the spectra shown in panel b. (**d**) Real part of the spectra for the 0.1-THz-irradiated sample, non-irradiated control and pure water at 25 °C. (**b-c**) All data are shown as means of four measurements. The measurement errors indicated by the shading are given by the standard deviation of the four measurements.

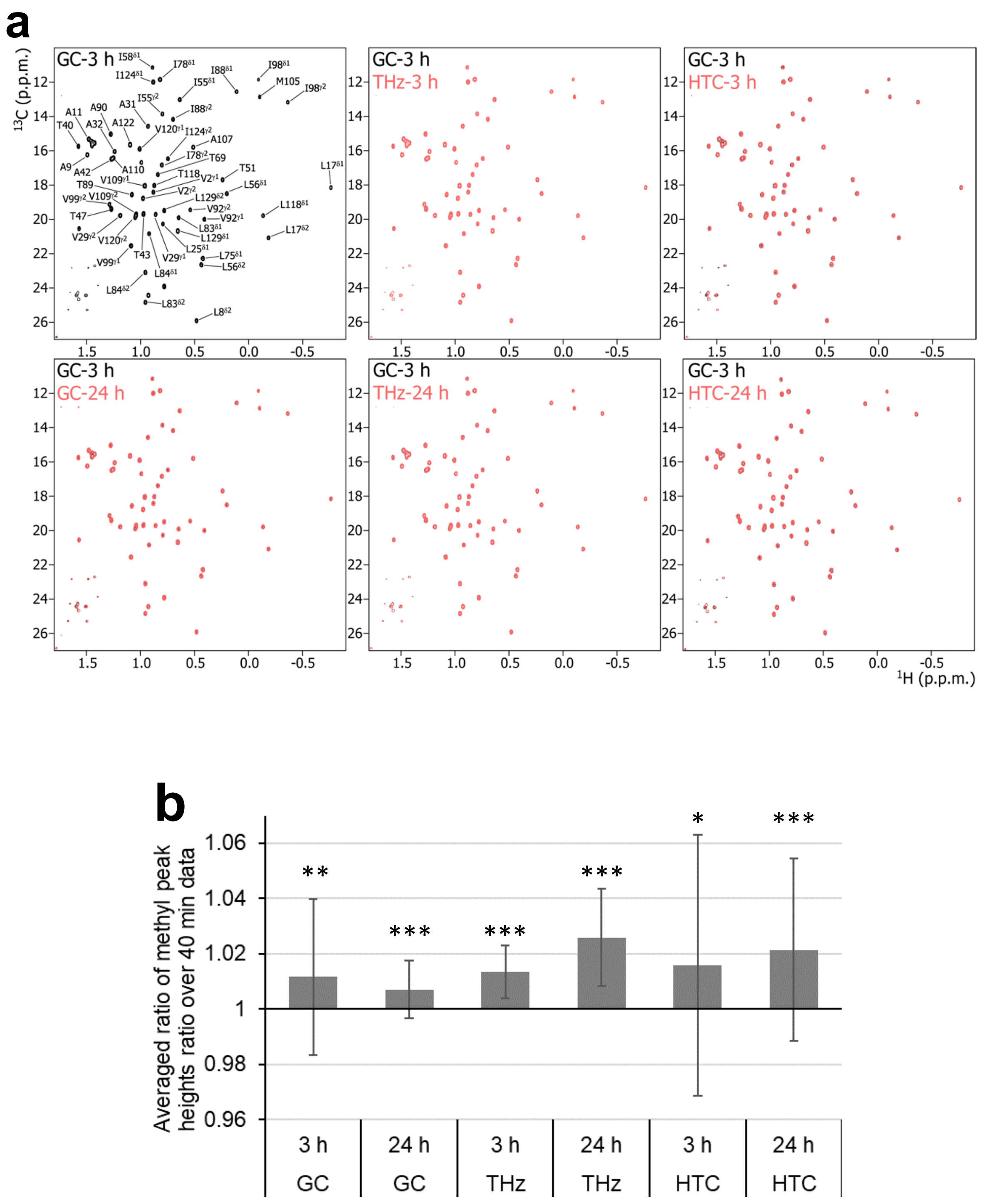

Supplementary Fig. 7. 2D ^{1}H-^{13}C HSQC spectra (methyl region) of the 9.1 wt% lysozyme solution. (a) Spectrum of the GC-3 h sample (upper left) with assignments transferred from reported data (Biological Magnetic Resonance Bank entry 4562). Spectra of other conditions, GC-24 h (bottom left), THz-3 h (upper middle), THz-24 h (bottom middle), HTC-3 h (upper right), and HTC-24 h (bottom right) are superimposed in red on that of GC-3 h (black). The black contours of GC-3 h are mostly masked below red contours of the superimposed spectra. **(b)** Averaged ratio of methyl peak heights for each spectra shown in panel a over those in the spectrum obtained 40 min after dissolution. The error bars indicate the standard deviations of ratio of all signals. *P*-values ($*P < 0.05 \times 10^{-3}$, $**P < 0.005$, $***P < 1 \times 10^{-5}$) of two-tailed t-test are shown for pairs with statistically significant differences to the spectrum obtained 40 min after dissolution. GC, general control sample; THz, irradiated sample; HTC, high-temperature control sample.

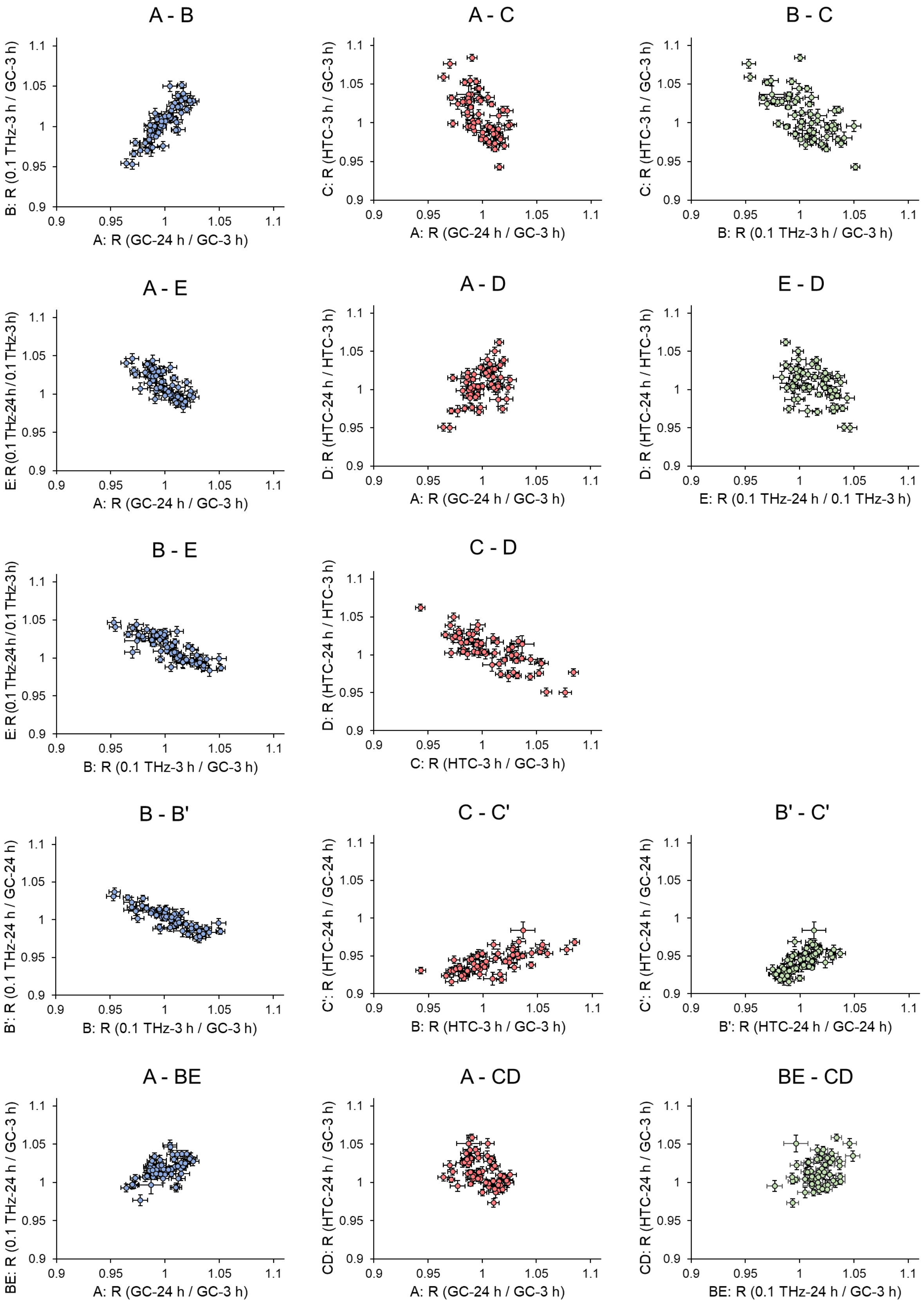
A - B
B: R (0.1 THz-3 h / GC-3 h)
A: R (GC-24 h / GC-3 h)
A - C
C: R (HTC-3 h / GC-3 h)
A: R (GC-24 h / GC-3 h)
B - C
C: R (HTC-3 h / GC-3 h)
B: R (0.1 THz-3 h / GC-3 h)
A - E
E: R (0.1 THz-24 h / 0.1 THz-3h)
A: R (GC-24 h / GC-3 h)
A - D
D: R (HTC-24 h / HTC-3 h)
A: R (GC-24 h / GC-3 h)
E - D
D: R (HTC-24 h / HTC-3 h)
E: R (0.1 THz-24 h / 0.1 THz-3 h)
B - E
E: R (0.1 THz-24 h / 0.1 THz-3h)
B: R (0.1 THz-3 h / GC-3 h)
C - D
D: R (HTC-24 h / HTC-3 h)
C: R (HTC-3 h / GC-3 h)
B - B'
B': R (0.1 THz-24 h / GC-24 h)
B: R (0.1 THz-3 h / GC-3 h)
C - C'
C': R (HTC-24 h / GC-24 h)
B: R (HTC-3 h / GC-3 h)
B' - C'
C': R (HTC-24 h / GC-24 h)
B': R (HTC-24 h / GC-24 h)
A - BE
BE: R (0.1 THz-24 h / GC-3 h)
A: R (GC-24 h / GC-3 h)
A - CD
CD: R (HTC-24 h / GC-3 h)
A: R (GC-24 h / GC-3 h)
BE - CD
CD: R (HTC-24 h / GC-3 h)
BE: R (0.1 THz-24 h / GC-3 h)
0.9
0.95
1
1.05
1.1

Supplementary Fig. 8. Correlation analysis for any pair of pathways shown in Fig. 6a. For each methyl signal, the ratio of signal intensity at the end point to that at the starting point of each pathway is plotted. The error bar of a ratio of i-th methyl site for a pathway X-Y is derived from the signal-to-noise ratio (SNR) as follows: $Error_{i,X-Y} = (1/SNR_{i,X} + 1/SNR_{i,Y})/\sqrt{2}$. GC, general control sample; THz, irradiated sample; HTC, high-temperature control sample.

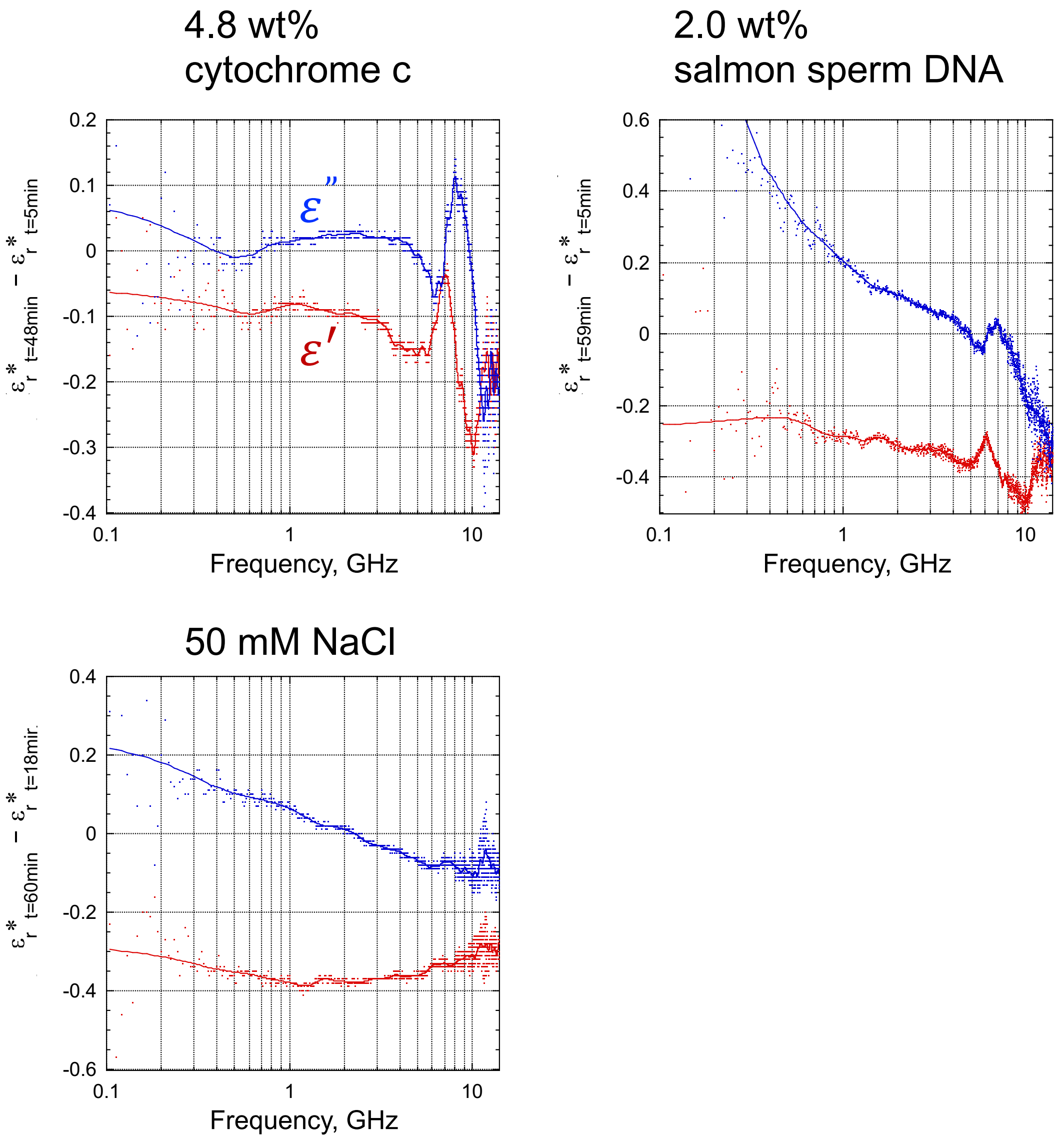

Supplementary Fig. 9. Difference in complex DR spectra before and after 0.1-THz irradiation. Real (red) and imaginary (blue) parts of the difference spectra are shown. Salmon sperm DNA and NaCl were dissolved in pure water. Cytochrome C was dissolved in a buffer containing 50 mM Tris-HCl (pH = 7.6). These solutions were prepared ~2 h before measurements. Cytochrome C and DNA were irradiated at 0.1 THz for 10 min (from t = 10

min to $t = 20$ min) and thereafter left at room temperature; except for these steps, the experimental procedure was identical to that for the lysozyme solution (see main text). DNA powder from salmon sperm and cytochrome C powder from horse heart were purchased from Fujifilm-Wako Pure Chemical (Cat. No. 049-31383) and Nacalai Tesque, Inc. (Cat. No. 10429-55), respectively, and used without further purification.

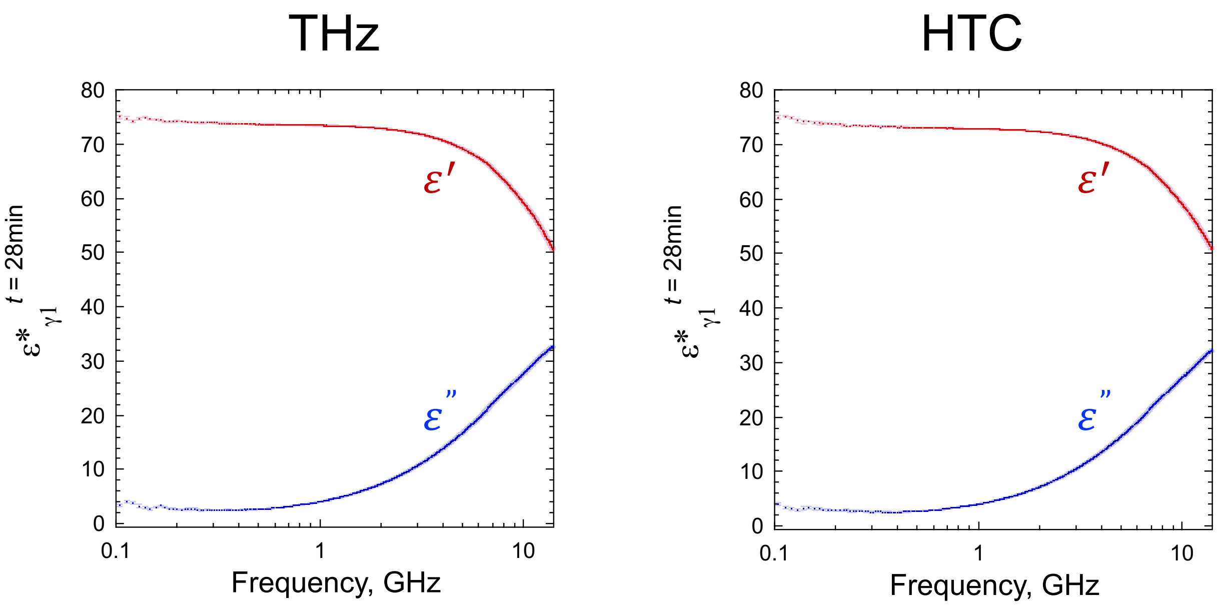

Supplementary Fig. 10. Noise due to the interference between the two electromagnetic fields was not observed during 0.1 THz irradiation. Complex DR spectra of 2.9 wt% lysozyme sample during 0.1 THz irradiation (left) and heating (right). The spectra were obtained by the time-lapse DR measurement (see Methods). The real (red) and imaginary (blue) parts of the spectra are shown. The means of five measurements ± standard deviations (red or blue shading) are shown.

Component	Δε	Δε, %	fc, GHz	τ, s/rad	Ref.
Protein (β)	7	8.7	0.01	1.6×10^{-08}	27
Hydration water (δ_1)	4	5.0	0.1	1.6×10^{-09}	27
Hydration water (δ_2)	1	1.2	4	4.0×10^{-11}	27
Slow bulk water (γ_1)	63	78.5	17	9.4×10^{-12}	27
Fast bulk water (γ_2)	1.26	1.6	600	2.7×10^{-13}	42
$\varepsilon(\infty)_{relax}$	4	5.0	-	-	42
$\varepsilon(s)$	80.3	100.0	-	-	-

Supplementary Table 1. Multiple relaxation components of the 9.1 wt% lysozyme solution modeled in our analysis.

Component	Δε	Δε, %	fc, GHz	τ, s/rad	Ref.
Protein (β)	3	3.7	0.01	1.6×10^{-08}	27
Hydration water (δ_1)	2	2.5	0.1	1.6×10^{-09}	27
Hydration water (δ_2)	0.5	0.6	4	4.0×10^{-11}	27
Slow bulk water (γ_1)	70	86.5	17	9.4×10^{-12}	27
Fast bulk water (γ_2)	1.4	1.7	600	2.7×10^{-13}	42
$\varepsilon(\infty)_{relax}$	4	4.9	-	-	42
$\varepsilon(s)$	80.9	100.0	-	-	-

Supplementary Table 2. Multiple relaxation components of the 2.9 wt% lysozyme solution modeled in our analysis.
